# Supplementary material for: Transcriptional Response of Streptomyces coelicolor to Rapid Chromosome Relaxation or Long-Term Supercoiling Imbalance
Source: Front Microbiol. 2019 Jul 11;10:1605. doi: 10.3389/fmicb.2019.01605 (PMC6637917; doi:10.3389/fmicb.2019.01605)
Supplement: Supplementary file 5 [file Table_5.pdf]

Table S5. Novobiocin and *topA*-upregulation overlapping genes

Gene	*Description*
sco0473	possible solute binding protein
sco0498	putative peptide monooxygenase
sco1905	hypothetical protein
sco2896	putative integral membrane protein
sco3451	hypotetical protein
sco4360	putative ABC transport system integral membrane protein
sco5285	Lon protease
sco5451	putative ABC transporter
sco5581	conserved hypothetical protein
sco5957	putative transport protein
sco6720	putative ABC transporter
sco7252	putative regulatory protein
sco7676	putative ferredoxin
